# A Case of Erythrodermic Psoriasis Successfully Treated With Ixekizumab Combined With Low-Dose Methotrexate to Ensure Sustained Clearance: A Case Report

**DOI:** 10.1155/crdm/8810497

**Published:** 2025-05-29

**Authors:** Jennifer Lavina Ngo, Lily Lyralin Tumalad, Patricia Anne Tinio, Rogelio Balagat

**Affiliations:** Department of Dermatology, Rizal Medical Center, Pasig, Philippines

**Keywords:** biologics, combination treatment, erythrodermic psoriasis, ixekizumab, methotrexate

## Abstract

Erythrodermic psoriasis (EP) is a severe type of psoriasis that requires immediate and effective treatment to prevent serious complications. Although recommended as first-line treatment for EP, conventional systemic disease–modifying antirheumatic drugs (csDMARDs) such as methotrexate and/or cyclosporine can sometimes be ineffective or undesirable, hence the use of biologics. However, in cases of refractory disease, biologics may be combined with methotrexate to boost efficacy and optimize outcomes without compromising safety and tolerability.

## 1. Introduction

Erythrodermic psoriasis (EP) is a rare and severe type of psoriasis that may be a consequence of poorly controlled disease or triggered by environmental factors such as infection, withdrawal of systemic steroids, severe emotional stress, and other systemic illnesses [1, 2]. Since EP may be potentially life-threatening, effective therapy must be administered as soon as possible to avoid the occurrence of systemic complications. Although recommended as first-line treatment for EP, conventional systemic disease–modifying antirheumatic drugs (csDMARDs) such as methotrexate and/or cyclosporine may be ineffective or not tolerated due to adverse effects [2]. Meanwhile, because of their selective cytokine inhibition, biologics have become a therapeutic option for EP by virtue of their efficacy and better safety profile [2, 3].

Among the biologics, interleukin-17 (IL-17) inhibitors such as secukinumab and ixekizumab have been found to be effective in achieving near-total clearance of EP, especially since IL-17 was found to be a key cytokine in EP [1–3]. However, in cases of refractory disease, biologics may be combined with methotrexate to boost efficacy and optimize outcomes without compromising safety and tolerability [4, 5]. Although utilized more frequently in rheumatoid arthritis, combining biologics with methotrexate has been proven to quickly provide complete skin clearance in patients with severe psoriasis [5]. The literature reports the efficacy of methotrexate in combination with TNF-alpha inhibitors or secukinumab for the treatment of moderate-to-severe psoriasis and psoriatic arthritis [4, 5]. On the other hand, ixekizumab in combination with methotrexate has only been documented in psoriatic arthritis [6].

This is the case of a pediatric patient with EP, who was resistant to methotrexate combined with cyclosporine but completely responded to the IL-17 inhibitor, ixekizumab, combined with low-dose methotrexate.

## 2. Case Report

**Figure 1:**
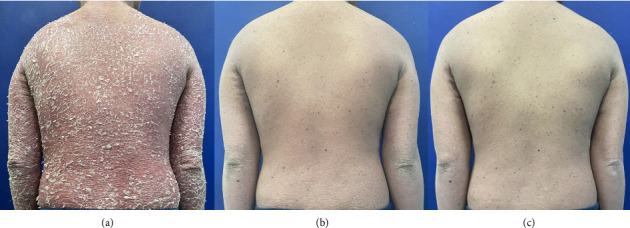
Physical exam. (a) Pretreatment: Generalized erythema with scaling involving > 90% BSA. (b) After 12 weeks of ixekizumab with low-dose methotrexate: PASI 75. (c) After 20 weeks of ixekizumab with low-dose methotrexate: PASI 100.

A 16-year-old Filipino male with Fitzpatrick skin Type IV sought consultation at the dermatology clinic of a tertiary hospital for generalized erythema with scaling affecting > 90% of his BSA (Figure 1(a)). He was a known case of chronic plaque psoriasis since age 11 with no other comorbidities. His paternal aunt had psoriasis. On further history taking, the patient was previously treated with methotrexate 10 mg weekly and folic acid supplementation, along with alternate courses of topical clobetasol and topical calcipotriol every 2 weeks until near remission was achieved. He was then eventually lost to follow-up and never went through phototherapy. At the time of consultation, the patient was afebrile, had stable vital signs, and weighed 34 kg with a normal body mass index (BMI). There was no bipedal edema. He had no signs of psoriatic arthritis and denied symptoms of such. The nails were normal.

The patient was diagnosed with EP and was treated simultaneously with methotrexate at a maximum of 17.5 mg per week (0.5 mg/kg) and cyclosporine at 2.75 mg/kg/day. After 6 months of continuous treatment with no reported adverse effects, a normal blood pressure, and laboratory indices (blood count with differential, liver enzymes, and creatinine level) within reference range, the patient still exhibited minimal improvement. Due to the refractory nature of his disease, the patient was started on ixekizumab 80 mg subcutaneous injections every 4 weeks in an off-label indication and dose for his age group in the Philippines. The biologic was given alongside low-dose methotrexate at 7.5 mg per week (0.2 mg/kg); cyclosporine was discontinued.

After 12 weeks of ixekizumab in combination with low-dose methotrexate, the patient achieved a psoriasis area and severity index (PASI) of 75 (Figure 1(b)). The patient received his last dose of ixekizumab at Week 20 in combination with methotrexate of the same dose with a good response at PASI 100, achieving complete skin clearance (Figure 1(c)) with good tolerability. There was no history of infection or derangement in laboratory indices (blood count and liver enzyme) throughout the combination treatment. Since discontinuation of ixekizumab, the patient remains lesion-free with low-dose methotrexate at Week 32, with no reported adverse effects.

Biologics have changed the treatment landscape of patients with psoriasis regardless of age, and although less studied as compared to adults, most biologics have been tested and approved for pediatric use [7, 8]. Due to the role of the IL-17 signaling pathway in psoriasis, the use of IL-17 inhibitors in the different subtypes of psoriasis has been most promising in achieving complete skin clearance at a rapid and efficient pace with a good safety profile [2, 8, 9].

Ixekizumab is an IL-17 human monoclonal antibody that was previously reported to be successful in treating EP [2, 3, 9], hence the decision to escalate treatment with this biologic to overcome the patient's resistance to methotrexate combined with cyclosporine. As of this writing, ixekizumab is Food and Drug Administration (FDA)–approved for the treatment of psoriasis in children at least 6 years of age elsewhere but has yet to be FDA–approved for the same age group in the Philippines [10]. On-label dosing for our patient's age and weight requires one 80 mg injection at Week 0 followed by one 40 mg injection every 4 weeks thereafter [11]. However, since ixekizumab only came in 80 mg autoinjector pens in the country, a half-based dosing of 40 mg was impossible. To optimize treatment, the patient was made to receive off-label dosing at 80 mg at the same frequency of every 4 weeks with the full consent of his mother. Meanwhile, the patient continued to receive low-dose methotrexate at 7.5 mg per week in combination with ixekizumab to prepare for the eventual discontinuation of the biologic drug due to financial constraints.

In a resource-constrained setting such as the Philippines, payments for treatment are largely out-of-pocket [12]. In the context of psoriasis, the use of biologics continues to remain a challenge due to the economic burden it brings to the government and to its payers. Government subsidies for effective but expensive treatment such as biologics are limited, forcing physicians to revert to a more affordable but not necessarily more effective treatment [12]. As a matter of fact, a multicenter study conducted in the Philippines revealed an underutilization of biologics in patients with moderate-to-severe psoriasis at 4% despite all patients achieving an improved outcome [13]. On the other hand, the same study reported that methotrexate was the most frequently prescribed systemic nonbiologic agent at 21% with only 68% achieving an improved outcome [13]. Similarly, a facility-based study conducted in the Southern Philippines revealed that only 6.1% of patients with psoriasis received biologics as opposed to 24.4% of patients who received methotrexate [14]. All these data consistently describe physicians' frequent use of methotrexate due to its low cost and accessibility in the Philippines.

Methotrexate has been found to have moderate to good efficacy in the treatment of psoriasis [15]. At low doses of 5–25 mg/week in psoriasis, its anti-inflammatory/immune-mediating effect renders a slow onset of action that requires a longer treatment administration before results become visible [15, 16]. Its acceptable safety profile and affordable cost make it a favorable treatment option in patients with psoriasis, especially in the Philippines. Our patient was seen at an end-referral government hospital, where he was able to receive limited doses of ixekizumab for free through a pharmaceutical-sponsored program. Since the patient will not be able to acquire the expensive biologics as maintenance, the authors decided to make use of both ixekizumab and methotrexate to maximize treatment outcomes and ensure sustained clearance even after discontinuation of the biologic drug. After 20 weeks of ixekizumab with low-dose methotrexate, the patient achieved complete skin clearance and ixekizumab was discontinued. The patient continues to remain on low-dose methotrexate at 7.5 mg per week as monotherapy for the past 12 weeks without recurrence of psoriatic lesions.

## 3. Conclusion

This case illustrates the effective and safe use of a biologic drug (ixekizumab) combined with a csDMARD (methotrexate) in the treatment of EP. This technique can also be used as a cost-saving measure that will allow the successful management of patients who have difficulty sustaining the maintenance treatment of expensive biologics. Due to its nature of being a slow starter, the concomitant use of low-dose methotrexate for at least 12 weeks provides a treatment reserve that will allow continuous efficacy once the biologic drug is discontinued. To the authors' knowledge, this is the first documented case utilizing the combination technique for economic purposes, rather than reasons of immunogenicity, without any negative sequelae. Furthermore, this case illustrates the strategies employed to overcome limitations in management secondary to finances in a resource-constrained setting such as the Philippines [17].

## Data Availability

The data that support the findings of this study are available from the corresponding author upon reasonable request.
